# An Analytical Study of Prostate-Specific Antigen Dynamics

**DOI:** 10.1155/2016/3929163

**Published:** 2016-11-13

**Authors:** Ernesto P. Esteban, Giovanni Deliz, Jaileen Rivera-Rodriguez, Stephanie M. Laureano

**Affiliations:** ^1^Physics Department, University of Puerto Rico-Humacao, José E. Aguiar Avenue, Road 908 Km 1.2, Humacao, PR 00791, USA; ^2^Biology Department, University of Puerto Rico-Humacao, José E. Aguiar Avenue, Road 908 Km 1.2, Humacao, PR 00791, USA

## Abstract

The purpose of this research is to carry out a quantitative study of prostate-specific antigen dynamics for patients with prostatic diseases, such as benign prostatic hyperplasia (BPH) and localized prostate cancer (LPC). The proposed PSA mathematical model was implemented using clinical data of 218 Japanese patients with histological proven BPH and 147 Japanese patients with LPC (stages T2a and T2b). For prostatic diseases (BPH and LPC) a nonlinear equation was obtained and solved in a close form to predict PSA progression with patients' age. The general solution describes PSA dynamics for patients with both diseases LPC and BPH. Particular solutions allow studying PSA dynamics for patients with BPH or LPC. Analytical solutions have been obtained and solved in a close form to develop nomograms for a better understanding of PSA dynamics in patients with BPH and LPC. This study may be useful to improve the diagnostic and prognosis of prostatic diseases.

## 1. Introduction

Most men in mature age experience an abnormal prostate growth (BPH), while others are faced with prostate cancer (PC). According to the World Cancer Research Fund International [1], prostate cancer is the second most common cancer in men worldwide. Regarding BPH, mortality in the last three decades has been declining, but its incidence as men get older increases [2, 3]. Further, a nonnegligible percentage of the aging men's population has both of these harmful diseases. In both of these prostatic diseases, prostate cancer cells as well as prostate normal cells secrete a protein called prostatic specific antigen (PSA), which is confined to the prostate gland, although some leak to the bloodstream.

In 1986, a clinical test to measure PSA in the blood was developed, and since then, it is used in screening PC. PSA rising levels in serum were associated with an increase of the prostate cancerous tumor size for larger prostate glands and with tumor volume in small, medium, or large prostates [4]. However, it was soon found out that there are other conditions such as prostatitis and BPH that can elevate PSA values. Even a temporal condition, such as a prostate biopsy, could also elevate PSA values. As a consequence, false positives and false negatives have made the PSA test controversial. False positives may lead to overtreatment and false negatives to metastasis, a cancer terminal stage (M category).

In the last three decades, many statistical and clinical studies of the relationships among PSA, PC, and BPH have been carried out. However, there are still many queries to answer. For instance, (i) what is the relationship among PSA, tumor's size, and age in patients with LPC and BPH? (ii) Is it possible to personalize the PSA test? (iii) Is it possible to mathematically model abnormal prostate growth? (iv) What is the relationship between PSA level value and LPC stage? Since numerous numerical, statistical, and clinical studies have not reached conclusive answers to the above queries, here in this paper we shall study PC and BPH using PSA analytical solutions of a nonlinear differential equation. These solutions are of interest because one wants to gain a greater understanding of PSA dynamics to influence treatment decisions of such prostatic diseases. Unfortunately, a search procedure to different databases yielded only a small number of papers describing PSA analytical studies [5]. In fact, we could not find any analytical PSA dynamics study in which BPH and LPC coexist simultaneously.

In 2001, Swanson et al. published an analytical model to study PSA dynamics of patients with prostate cancer [5]. In doing so, Swanson et al. [5] assumed (i) a prostate cancer tumor has an exponential growth and (ii) the PSA contribution of normal prostate cells to the PSA total serum is negligible. Swanson's et al. mathematical model [5] allowed the understanding of a medical anomaly; that is, why patients with an aggressive prostate cancer tumor could have low PSA value levels? Also, Swanson's model [5] showed a good agreement with xenografts of human prostate carcinoma implanted in mice. Swanson's et al. study [5] also showed how mathematical models could be useful for a better understanding of PC PSA dynamics.

In this paper, we present analytical solutions of PSA dynamics for patients with BPH and LPC. Contrary to Swanson et al. [5], we have not neglected the PSA contribution of normal prostate cells.

Three cases are considered, namely, PSA dynamics for patients with LPC and BPH and for those with both LPC and BPH.

## 2. Patients and Methods 

As Swanson et al. [5] pointed out, PSA dynamics for normal and cancer cells satisfy the following nonlinear differential equation:

(1) $$\begin{array}{c} \frac{dp}{dt}=\beta_{c}V_{c}+\beta_{h}V_{h}-\gamma p, \end{array}$$

where *p* is the total serum PSA at time *t* and *V*_*h*_ and *V*_*c*_ are functions representing the evolving on time of normal and cancerous prostate volumes, respectively. PSA is metabolized by the blood at a constant rate *γ*, and it is leaked to the bloodstream at rates *β*_*c*_ and *β*_*h*_, for cancerous and normal cells, respectively.

Swanson's et al. [5] assumed that a cancerous tumor grows exponentially. Clinical studies by Friberg and Mattson [6] have confirmed the exponential model for tumor growth. Swanson et al. [5] neglected the PSA contribution of prostate normal cells to the total serum PSA on basis that the secretion rates satisfy the inequality *β*_*h*_ ≪ *β*_*c*_. Although this assumption may be valid for a normal prostate size, it fails for patients with both harmful conditions BPH and a prostate cancer tumor.

### 2.1. PSA Dynamics for Patients with Normal Size Prostate and LPC

In this case, *V*_*c*_ = *V*_*o*_ *e* ^*ρ*_*c*_(*t* − *t*_*o*_)^, where *V*_*o*_ is the tumor's size at time *t*_*o*_ and *ρ*_*c*_ is the tumor's growth rate. The PSA contribution by normal prostate cells (*β*_*h*_ = 0) is neglected, and thus the particular solution of (1) can be written as

(2) $$\begin{array}{c} p_{c}=p_{o}e^{-\gamma \left(t-t_{o}\right)}+\frac{\beta_{o}V_{o}}{\gamma +\rho_{c}}\left(\;e^{\rho_{c}\left(t-t_{o}\right)}-e^{-\gamma \left(t-t_{o}\right)}\;\right). \end{array}$$

The total PSA serum leaked to the blood by the prostate cancerous cells (*p*_*c*_) is given by (2), where *ρ*_*o*_ is the PSA value measured at time *t*_*o*_. To make contact with the literature, we set *t*_*o*_ = 0 and *p*_*o*_ = 0 in (2). We obtain

(3) $$\begin{array}{c} p_{c}=\frac{\beta_{c}V_{o}}{\gamma +\rho_{c}}\left(\;e^{\rho_{c}t}-e^{-\gamma t}\;\right). \end{array}$$

Equation (3) was first obtained by Swanson's et al. [5]. However, notice that there is a misprint on (1) of Swanson et al. [5].

A prostate cancer tumor could grow fast or slowly. This means that a given cancerous prostate tumor volume could be reached in a short or long time. This will depend in this study on the *ρ*_*c*_ value. Recent studies [7] have shown that the prostate cancer tumors cannot evolve from low to high grade, and therefore *ρ*_*c*_ could be considered constant, although of a different value for a slow or aggressive prostate cancer. Therefore, inside the prostate we may have prostate tumors with different growing capabilities, for example, stage T2a, when a tumor is in one-half or less of only one side (left or right) of the prostate, and in stage T2b in which the tumor is in more than half of only one side (left or right) of the prostate. In this paper, we shall consider that tumors associated with T2a and T2b stages will grow to a maximum volume of 4.5 cm^3^ and 12.5 cm^3^, respectively.

For a slowly growing prostate cancer tumor (3) can be written as

(4) $$\begin{array}{c} p_{cs}=\frac{\beta_{c}V_{c}}{\gamma }. \end{array}$$

Equation (4) suggests that the PSA value (*p*_*cs*_) and the cancer tumor volume (*V*_*c*_) will be strongly correlated. This result obtained using a mathematical procedure was found also in clinical studies [8]. Moreover, using (4) it is possible to estimate the value of *β*_*c*_ when clinical data for the PSA and the cancer tumor volume is provided.

PSAD (PSA density), PSAV (PSA velocity), and PSAA (PSA acceleration) can easily be calculated from (4). We obtain

(5) PSAD=βcγ,

(6) PSAV=ρcpc,

(7) PSAA=ρc2pc.

Equations (6)-(7) suggest that for a slowly growing prostate tumor PSAV and PSAA correlate with the value of PSA. This could mean that PSAV is not a better cancer marker than the PSA. Vickers et al. [9] reached the same conclusion. These researchers did not find evidence to support that men with higher PSAV should be biopsied in the absence of others indicators. The value of *ρ*_*c*_ for slowly growing prostate tumor can be estimated by solving (6), Thus,

(8) $$\begin{array}{c} \rho_{c}=\frac{0.693}{T_{DT}}, \end{array}$$

where *T*_*DT*_ is the PSA doubling time.

PSA doubling times (*T*_*DT*_) for prostate cancer stages can be obtained from the literature and therefore, the corresponding value of *ρ*_*c*_ can be estimated from (8).

### 2.2. PSA Dynamics in Patients with a BPH Condition

We have found using clinical data published in the literature [10, 11] that men's prostate enlargement as they aged can be modeled by a cubic equation on time. Thus, it will be assumed in (1) that *V*_*h*_ = *a* + *a*_1_ *t* + *a*_2_ *t* ^2^ + *a*_3_ *t* ^3^, as also suggested by Xia et al. [12]. The solution of (1) after plug in *β*_*c*_ = 0 can be written as

(9) $$\begin{array}{c} p=\frac{1}{\gamma^{4}}\left(\;C_{1}+C_{2}\gamma +C_{3}\gamma^{2}+C_{4}\gamma^{3}+C_{5}\gamma^{4}\;\right), \end{array}$$

where

(10) C1=6βhe−γt−to−1d3Vhdt3,C2=βhγd2Vhdt2−e−γt−tod2Vhdt2t→to,C3=βhγ2dVhdt+e−γt−todVhdtt→to,C4=βhγ3Vh+e−γt−toVht→to,C5=poγ4e−γt−to,

when *γ* ≫ 1; (9) reduces to *p* ≈ (*β*_*h*_/*γ*)*V*_*h*_. Thus, for BPH, PSA strongly correlates with the prostate volume, a fact that is also cited in the literature [13] and here for the first time analytically proved. PSA density (PSAD), PSA velocity (PSAV), and acceleration (PSAA) can also be easily obtained from (9)-(10). Also, the *γ* parameter can be found from (1) by setting *V*_*h*_ = 0. Clinically, this means the removal of the prostate using a surgical procedure called radical prostatectomy. In this case

(11) $$\begin{array}{c} \gamma =\frac{0.693}{\tau_{1/2}}, \end{array}$$

where *τ*_1/2_ is the PSA half-life.

### 2.3. PSA Dynamics in Patients with PC and BPH

In this case, a patient has both LPC and BPH harmful conditions. Thus in (1), both values of *β*_*c*_ and *β*_*h*_ are different of zero. Therefore, by plugging the analytical expressions of *V*_*h*_ and *V*_*c*_ in (1), we can solve it in a closed form without neglecting any term. We obtain

(12) p=1γ4γ+ρcC1+C2γ+C3γ2+C4γ3+C5γ4+C6γ5,

(13) C1=−d3Vhdt3βhρc1−e−γt−to,

(14) C2=βhρcd2Vhdt2−d2Vhdt2t→toe−γt−to−βhd3Vhdt31−e−γt−to,

(15) C3=βhd2Vhdt2−e−γt−tod2Vhdt2t→to−βhρce−γt−todVhdtt→to−dVhdt,

(16) C4=βhρcVh−dVhdt+βhdVhdtt→to−ρcVhto·e−γt−to,

(17) C5=βhVh+poρc−βcVo−βhVhtoe−γt−to+βcVoeρct−to,

(18) C6=poe−γt−to.

Notice the coupled terms in (13)–(18). This means that the total PSA serum for patients with BPH and LPC cannot be found just by adding the respective particular solutions for LPC and BPH. Also, from (12) it can be seen that the *γ* parameter may play a major role in PSA dynamics. This is because when *γ* ≫ 1, the only relevant coefficients will be given by (17)-(18).

## 3. Results and Discussion

### 3.1. PSA Dynamics for Japanese Patients with LPC

Next, we shall focus in palpable LPC, in particular, prostate cancer stages T2a and T2b. Here, following [14], in a T2b stage, the maximum prostate tumor volume is 12.5 cm^3^. For tumors in a T2a stage, a maximum prostate tumor volume of 4.5 cm^3^ is assumed.

Tumor volume progression (*V*_*c*_) is a function of *ρ*_*c*_, the tumor's growth rate. The values of *ρ*_*c*_ for LPC were found using (8) and the PSA doubling times (*T*_*DT*_) values for T2a (110 months) and T2b (33.3 months) as given in [15]. Therefore, the values of *ρ*_*c*_ are 0.0756/year and 0.2497/year for T2a and T2b, respectively.

The PSAD clinical values for Japanese patients with proven T2a and T2b given by Furuya et al. [8] are 0.30 (ng/mL)/cm^3^and 0.54 (ng/mL)/cm^3^, respectively. Now, using the fact that PSA half-life is about 3 days, we can use (5) and (11) and the PSAD values obtained by Furuya et al. [8] to estimate the values of *β*_*c*_. We obtain, for T2a and T2b, 25.29 (ng/mL/year)/cm^3^ and 45.52 (ng/mL/year)/cm^3^, respectively.

**Figure 1:**
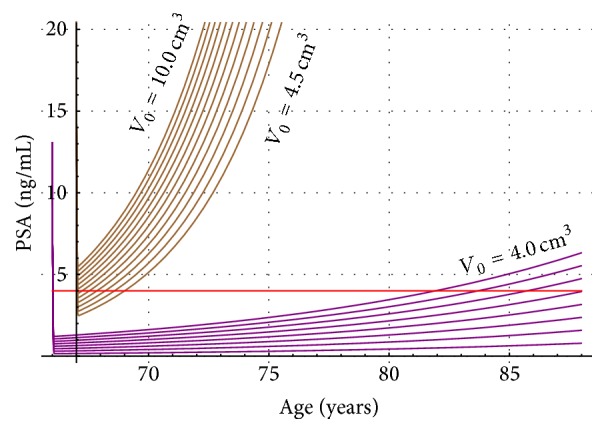
PSA versus age for prostate cancer patients stage T2a (purple) and T2b (brown). Each curve is associated with an initial tumor volume. It begins at 0.5 cm^3^ (T2a) or 4.5 cm^3^ (T2b) increasing 0.5 cm^3^ bottom up to reach a maximum tumor volume of 4.0 cm^3^ (T2a) or 10.0 cm^3^ (T2b). The red line shows the PSA standard cut-off value (4 ng/mL).

**Figure 2 fig2:**
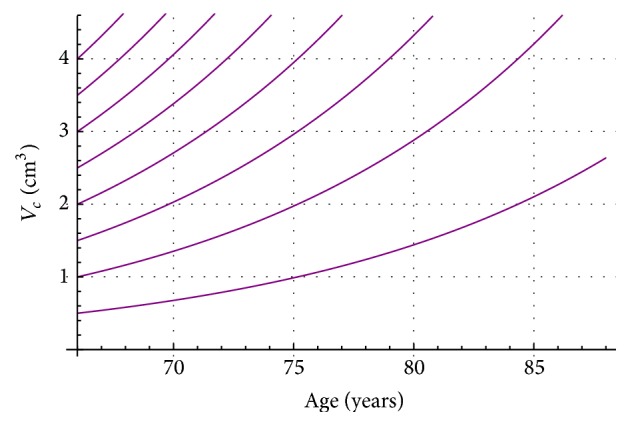
Tumor progression versus age, for stage T2a for Japanese prostate cancer patients.

**Figure 3:**
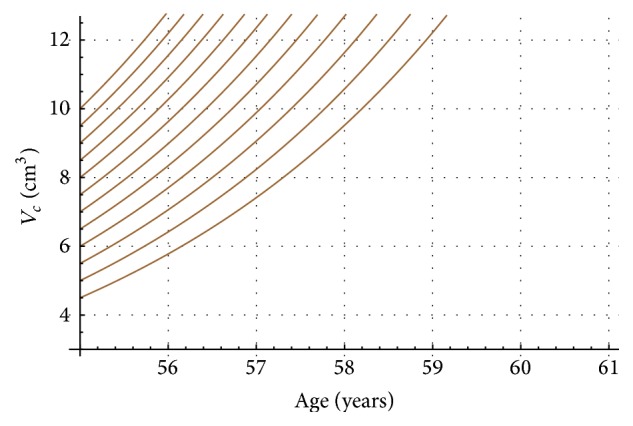
Tumor progression versus age, for stage T2b for Japanese prostate cancer patients.

In what follows, we shall focus on Japanese prostate cancer patients at LPC stages T2a and T2b. Initial conditions (age and PSA were taken from Table 1 in [8]) for both patients are T2a (66 years, PSA = 13.1 ng/mL, *β*_*c*_ = 25.29 (ng/mL/year)/cm^3^) and T2b (67 years, PSA = 21.7 ng/mL, *β*_*c*_ = 45.52 (ng/mL/year)/cm^3^). Here we shall point out that of course initial conditions could vary in other patients, but this situation can be easily taken care in our computational program. Next, in Figure 1, two nomograms (T2a in purple and T2b in brown) show PSA progression on age for different initial tumor volumes. T2a goes from 0.5 cm^3^ (lowest curve) to 4 cm^3^ (highest curve), and T2b goes from 4.5 cm^3^ (lowest curve) to 10 cm^3^ (highest curve). Figure 1 must be complemented with Figures 2 and 3. Figures 2 and 3 show for stages T2a and T2b the tumor volume progression for different initial tumor volumes values (T2a from 0.5 cm^3^ to 4.0 cm^3^ and T2b from 4.5 cm^3^ to 10 cm^3^).

### 3.2. PSA Dynamics for Japanese Patients with BPH

In this case, we shall use the clinical data published in [10] of 218 Japanese patients with histological proven hyperplasia. As we pointed out before, the enlargement of the prostate volume as a man aged can be modeled by a cubic equation on time. We found a very good fit between clinical data (prostate volume versus age) for BPH Japanese patients taken from [10] and a cubic equation on time *V*_*h*_ = −716.375 + 31.550*t* − 0.435*t* ^2^ + 0.002*t* ^3^.

**Figure 4:**
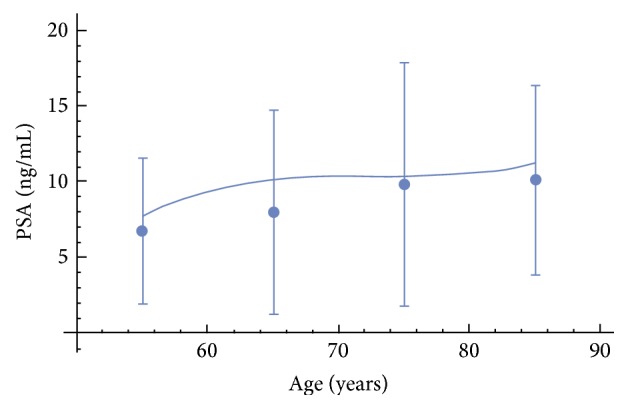
Clinical data for PSA for BPH Japanese patients (dots) as compared to predicted theoretical PSA values.

Now, using the clinical data published in table of [10] we have plotted (not showed here because editorial rules limit the number of figures) PSA versus prostate volume. A linear regression analysis gives a slope value of *β*_*h*_/*γ* = 0.22 (ng/mL)/cm^3^. Thus, for BPH Japanese patients, the *γ* and *β*_*h*_ parameters values are estimated to be 84.3 1/year and 18.9 (ng/mL/year)/cm^3^, respectively. Now, from (9) we can plot PSA versus age (years) in Figure 4.

PSAV can be also easily obtained from (9). For *γ* ≫ 1, PSAV can be approximated by a quadratic equation. Contrary to the expected that is a PSAA always positive, our results suggest that for BPH Japanese patients the PSAV values will be decreasing (PSAA negative) to reach zero at about 73 years old and then will increase (PSAA positive).

### 3.3. PSA Dynamics for Japanese Patients with BPH and PC

**Figure 5:**
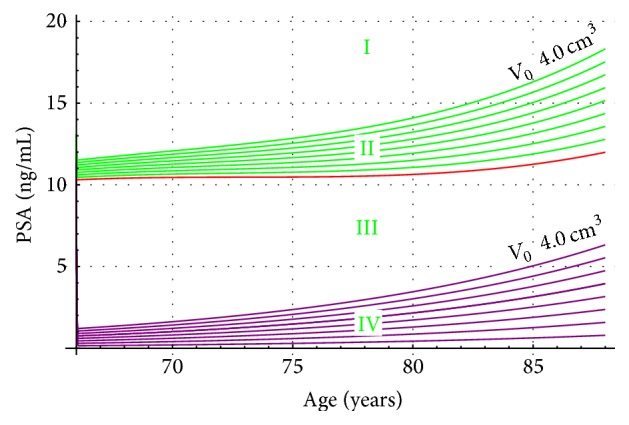
PSA level values for Japanese prostate cancer patients (T2a, purple) as compared to hypothetical BPH and prostate cancer Japanese patients (green). The red line is the PSA value of the BPH patients obtained from Figure 4.

**Figure 6:**
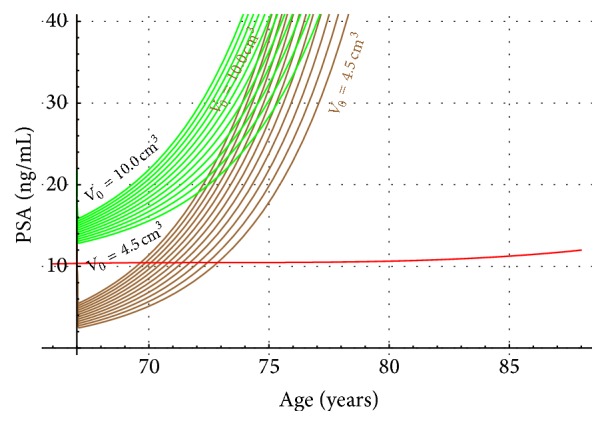
PSA level values for Japanese prostate cancer patients (T2b, brown) as compared to hypothetical BPH and prostate cancer Japanese patients (green). The red line is the PSA value of the BPH patients obtained from Figure 4.

Two nomograms (Figures 5 and 6) summarize our results for hypothetical Japanese patients with both harmful conditions BPH and LPC. In Figure 5, T2a (in purple) go from bottom up incrementing from an initial tumor volume of 0.5 cm^3^ to 4 cm^3^. The PSA contribution of Japanese patients with a proven BPH is plotted in red. The PSA dynamics of hypothetical Japanese patients with PC (T2a stage) and BPH is shown (in green). For a comparison, we also plot in Figure 6 (in brown) PSA versus age, for Japanese patients with PC (T2b stage). In Figure 6, the PSA dynamics of hypothetical Japanese patients with prostatic diseases PC (T2b stage) and BPH is also shown (in green).

Nomograms in Figures 1 [Fig fig2]–3 convey new information for the management of LPC T2a (purple) and T2b (brown). For instance, let us assume prostate cancer patients* A* (75 years old) and* B* (69 years old) both take the PSA test, with a result of 4 ng/mL for both of them (red line in Figure 1). In the standard procedure this PSA value of 4 ng/mL will probably induce the physician to recommend to both men to take a biopsy. Nowadays, the PSA test does not provide any information about tumor size, stage, or tumor and PSA progression on time. However, in this new PSA test interpretation, from Figure 1, we can recognize immediately that prostate cancer tumor in patient A cannot be staged as T2a or as T2b. However, from Figure 1, prostate cancer tumor in patient B must be staged as T2b. Also, the nomogram in Figure 1 shows an initial tumor volume of 4.5 cm^3^ when patient B was 67 years old. Next, Figure 3 allows following the tumor progression on time for patient B and predicts that two years later when patient B is 69 years old the tumor prostate cancer will be 6.5 cm^3^.

In Figures 5 and 6, nomograms describe hypothetical Japanese patients with BPH and LPC stages T2a (purple) and T2b (brown). Notice, in Figure 5, the first curve (purple) associated with stage T2a begins from the bottom and it is associated with an initial tumor volume of 0.5 cm^3^, increasing up each time 0.5 cm^3^ until 4.0 cm^3^. The corresponding eight curves in green show PSA dynamics for Japanese patients with both conditions BPH and LPC (stage T2a and BPH).

Figure 5 shows four regions (I, II, III, and IV). The first one is above the last green curve (4 cm^3^). Region II is the green zone bounded by the red line. Region III is bounded by the red line and the purple (4 cm^3^). In purple, the IV region is shown. In region I, neither T2a stage nor BPH are possible. All PSA values for T2a and BPH Japanese patients are in region II. Most likely PSA values for BPH Japanese patients should be in region III. Finally, in region IV PSA values of Japanese patients associated with a T2a stage will be confined. Figure 6 provides similar information as Figure 5 except that in Figure 6 it is assumed that the stage for LPC is T2b. Each of the green curves is associated with an initial tumor volume value, which goes up from 4.5 cm^3^ to 10 cm^3^. Notice also that in Figure 6 there is overlapping of the region T2b (Brown) and T2b and BPH (green).

A comparison between Figures 5 and 6 shows that the PSA values are higher in T2b Japanese patients as compared to those staged as T2a. This fact was also found in clinic and reported in [16].

## 4. Conclusions 

In this paper we have first developed a theoretical framework to study PSA dynamics for BPH and prostate cancer patients. This analytical study then was applied to obtain nomograms for a better understanding of the relationship among PSA and tumor volume in Japanese men with proven BPH or proven prostate cancer. This novel approach which does not neglect PSA contribution due to BPH may provide new information useful for a better diagnostic and prognosis of prostatic diseases. As an example of our mathematical procedures and analytical results we have developed nomograms for localized prostate cancer and also for patients with both prostatic diseases (BPH and LPC).

Although there are other nomograms published in the literature, none of these provide a relationship among PSA, age, and tumor volume as described in this paper. Also, we are aware that the results obtained in this PSA study for Japanese prostate cancer patients may be different for men of other ethnicities. It is our hope that this research will induce prostate cancer researchers to study new ways to interpret the PSA test.
